# Emerging Needs and Viability of Telepsychiatry During and Post COVID-19 Era: A Literature Review

**DOI:** 10.7759/cureus.16974

**Published:** 2021-08-07

**Authors:** Jayasudha Gude, Rashmi V Subhedar, Michelle H Zhang, Pratik Jain, Jatminderpal Bhela, Fariha Bangash, Nikhila Veluri, Ya-Ching Hsieh, Batool Z Sheikh, Mansi R Shah, Zeeshan Mansuri, Kapil Aedma, Urvish K Patel, Tapan Parikh

**Affiliations:** 1 Psychiatry, Northwell Health, Zucker Hillside Hospital, New York, USA; 2 Family Medicine, Mc Master University, Hamilton, CAN; 3 Psychological & Brain Sciences and Biology, Johns Hopkins University, Baltimore, USA; 4 Psychiatry, State University of New York Upstate Medical University, Syracuse, USA; 5 Psychiatry, Case Western Reserve University/Metrohealth system, Cleveland, USA; 6 Psychiatry, American University of Integrative Science School of Medicine, St. Michael, BRB; 7 Public Health, Icahn School of Medicine at Mount Sinai, New York, USA; 8 Psychiatry, Dow University of Health Sciences, Karachi, PAK; 9 Psychiatry, Brookdale University Hospital Medical Center, New York, USA; 10 Psychiatry, The Wright Center for Graduate Medical Education, Scranton, USA; 11 Psychiatry, Boston Children’s Hospital/Harvard Medical School, Boston, USA; 12 Psychiatry, Unity Point, Peoria, USA; 13 Public Health and Neurology, Icahn School of Medicine at Mount Sinai, New York, USA; 14 Psychiatry, Northwestern University Feinberg School of Medicine, Chicago, USA

**Keywords:** tele psychiatry, covid 19, technology, viability of telepsychiatry, tele mental health, virtual clinics, communication

## Abstract

The coronavirus disease 2019 (COVID-19) pandemic has resulted in nationwide stay-at-home orders in an effort to slow the spread severely impacting the healthcare sector. Telepsychiatry provides a platform bridging the gap through advanced technologies connecting mental health providers and patients who need their services, overcoming previous barriers of great distances, lack of transportation, and even time constraints. The most obvious benefit is increased accessibility to mental healthcare, especially in underserved and remote areas where there is no easy access for in-person care. It is important to note that benefits are not limited to patients, but also allow clinicians greater flexibility in scheduling and reduced practice overhead costs, both of which aid with physician burnout and burden. Telepsychiatry during COVID-19 provides its own unique advantages over in-person visits. The risk of exposure to healthcare workers and patients receiving care is reduced, allowing immunocompromised patients to receive much-needed psychiatric care. Without the need to meet in person, self-isolating psychiatrists can still provide care, decreasing strain on their co-workers. Although telepsychiatry is relatively new, it has already exhibited considerable success in its effectiveness at treating psychiatric conditions and widespread corollary benefits. Telepsychiatric consults may be carried out synchronously and asynchronously, each having benefits and setbacks. Different mobile application interventions have been explored, which are available for the purpose of both monitoring/assessing patients and/or providing treatment. The scope of conditions these applications address is broad, from anxiety disorders to schizophrenia to depression. As promising and beneficial telepsychiatry may seem, it is necessary to recognize that building the program can be challenging. It involves adapting to new methods in medicine. We highlighted barriers to general telepsychiatry, the most prominent being technological literacy of both physician and patient, and possible negative effects of eliminating the in-person patient-doctor interaction.

## Introduction and background

The recent coronavirus disease 2019 (COVID-19) pandemic has resulted in nationwide stay-at-home orders in an effort to slow the spread of the disease severely impacting many industries, including the healthcare sector [1]. As hospitals and healthcare providers pause non-emergency services and elective procedures, many patients are unable to receive care. In fact, 45% of U.S. adults reported they or someone in their household had deferred medical care due to the coronavirus pandemic [2]. Although the lack of these services may seem insignificant given their non-emergent status, they can still prove detrimental to an individual’s well-being, especially when it comes to mental health. With patients being unable to see their psychiatrists for in-person sessions, it is imperative that alternative methods be explored in an attempt to bypass this obstacle.

As the prevalence of mental health disorders continues to increase among young adults, there is an imminent need for new and innovative ways to meet the populations' demands of effective delivery of psychiatric care [3]. In addition to the challenges introduced by COVID-19, there is an existing disparity regarding access to healthcare; 3.6 million Americans are unable to receive medical care due to a lack of transportation [4]. Telepsychiatry offers a prospective solution to all the aforementioned problems, promising a delivery method that is not only much easier to access but can adapt to match the demand whilst abiding by social distancing policies in place [5].

Telepsychiatry provides a virtual platform for the matching and communication between mental health providers and the patients who need their services, overcoming previous barriers of great distances, lack of transportation, and even time constraints. Many mental health conditions require outpatient follow-up and medical management. Given that, telepsychiatry has the potential to increase access to care while improving waiting times and reducing costs and the need for hospitalization [6]. 

This review examines the methods and limitations of telepsychiatry that are currently available and provides valuable insight into the outcomes of populations that are currently being implemented in telepsychiatry. It is important to note that telepsychiatry is not a cure-all solution. Despite the encouraging benefits, there are many questions to be considered, such as: Will the patient feel as connected with the provider as in-person visits? Can the same trust and emotional bond be forged over a virtual session? Psychiatry is different from other medical specialities where treatment is often a prescription or a one-time procedure. Ninety percent of the diagnosis and fifty percent of treatment involve direct communication between patient and psychiatrist, so it is imperative to address how telepsychiatry may modify or change the physician-patient relationship.

## Review

With the COVID-19 pandemic came a dramatic shift of the U.S. healthcare system as traditional medicine transitioned from in-person visits to telemedicine instead. Without a vaccine or standardized effective treatment options, social distancing procedures and stay-at-home orders have become the primary forms of intervention. Given that, clinicians, healthcare workers, and insurers alike have been forced to adapt rapidly in order to provide an alternative to in-person care, turning to telemedicine. This surge in telemedicine has largely been focused on triage screening patients, much of which has been related to physical symptoms pertaining to COVID-19 [7,8]. What has been neglected amidst this pandemic, though, is the delivery and availability of mental healthcare. Whether it be the detrimental effects of social isolating, the panic that comes from losing one’s job in a period of economic instability, or simply the stress that comes from living during such uncertain times, it is clear that mental health is more important than ever. During this viral outbreak, there has been a huge surge in symptoms of depression, anxiety, insomnia, and acute stress, whether it be healthcare providers or the general public [9-11]. As the prevalence of mental health disorders continues to increase at a startling rate, it is pertinent to turn to telepsychiatry as a means of increasing access to mental healthcare.

Before diving into the benefits, limitations, and methods of implementation of telepsychiatry, it is important to gain a thorough understanding and definition of telepsychiatry (Table 1). Specifically, what makes it different from telemedicine? Telemedicine is described as the remote assessment, diagnosis, and treatment of patients through the use of telecommunication audiovisual technology. This includes, but is not limited to, consultations over the phone, video calls or text messaging, digital transmission of medical imaging, and remote diagnoses. Telepsychiatry is a specific application of telemedicine within the speciality of psychiatry and involves making psychiatric diagnoses and administering psychiatric treatment or therapy through technological means. The history of telemedicine is long and extensive, with the first use of telemedicine being described in The Lancet in 1879 as the transmission of medical knowledge and conducting medical appointments through the telephone [12]. However, telepsychiatry was not documented until nearly a century later, in the 1950s, when the Nebraska Psychiatric Institute utilized videoconferencing for consultations, educational endeavours, and training for patients [13]. 

**Table 1:** Comparison of traditional telemedicine with telepsychiatry

Telemedicine	Telepsychiatry
Remote assessment, diagnosis, and treatment of patients through the use of telecommunication audiovisual technology.	Application of telemedicine within the specialty of psychiatry.
Use of telemedicine was first described in The Lancet in 1879.	Related terms are telemental health and telepsychology.
	Administration of psychiatric treatment or therapy via technological communication.
	First use of videoconferencing in psychiatry was in the 1950s at the Nebraska Psychiatric Institute.

Although telepsychiatry is a newer branch of telemedicine, the potential benefits of implementation are extensive and far-reaching (Table 2). The increased accessibility to mental healthcare especially for those in underserved and remote areas seems to benefit maximally; additionally, transportation times and expenses are reduced along with base patient costs. These benefits result in lower attrition rates of patients, along with increased patient satisfaction with care [14-16]. It is important to note that benefits are not limited to patients, but also allow clinicians greater flexibility of scheduling and reduces practice overhead costs, both of which can aid with physician burnout and burden. Telepsychiatry during COVID-19 provides its own unique advantages over in-person visits. The risk of exposure to both healthcare workers and patients receiving care is greatly reduced, allowing immunocompromised patients to receive much-needed psychiatric care. Without the need to meet in person, self-isolating psychiatrists can still provide care, decreasing strain on their co-workers. Patients who have tested positive for coronavirus will still be able to meet with their psychiatrists.

**Table 2:** Pros and cons of telepsychiatry

Telepsychiatry Implementation and Potential Barriers and Benefits [14-16]	
Barriers	Benefits
Adapting to novel practice model and technology	Accessibility to specialized mental health care in underserved and remote areas
Interruption of the in-person patient-doctor interaction	Expand outreach, delivery, and efficiency of mental health practice
Liabilities and malpractice coverage concerns	Reduction in transportation time and expenses
Technology costs and security	Low attrition rate of patients
Billing and reimbursement constraints	Relieve physician burnout and increase well-being
Out-of-state physician licensing and credentialing issues	Improved patient satisfaction
Obtaining vital signs and necessary physical examinations, although the physical examination is less of a barrier in telepsychiatry compared to traditional telemedicine except for the evaluation of movement disorder or extrapyramidal side effects (EPS)	Improve clinical workflow and increase practice efficiency
Concerns about patient confidentiality and security	Reduce practice overhead
Need for psychiatrists as well as patients to have a basic level of technological literacy; both parties will need to adapt to the technology	Reduce patient costs
Patients with moderate to severe mental health illness might be reluctant to discuss their problems over teleconsultation services which can be a huge barrier.	Reduce emergency room visits
	Early detection allowing for preventative measures
	Flexibility of scheduling
	During COVID-19:
	Minimize risk of exposure to healthcare workers, as well as patients receiving care
	Immunocompromised patients will still be able to receive psychiatric care
	Self-isolating psychiatrists can still provide care; prevents increasing strain on remaining co-workers

Within the branch of telepsychiatry, one can dissociate between synchronous and asynchronous telepsychiatry, each having its own benefits and setbacks. Synchronous telepsychiatry requires the presence of both the patient and the psychiatrist at the same time, engaging in real-time communication. Asynchronous telepsychiatry, on the other hand, can be thought of as the store-and-forward transmission of medical data, as well as clinical diagnosis, treatment, and recommendations [17,18]. For an extensive comparison of the relative benefits and setbacks, refer to Table 3. Given the varying advantages, we recommend that the use of either method be situational; it is a decision that should be made largely depending on the patient-provider relationship and what would optimize individual patient care. 

**Table 3:** Comparison of synchronous vs asynchronous telepsychiatry

Synchronous Telepsychiatry [17-24]	Asynchronous Telepsychiatry [25-28]
Consists of live videoconferencing and direct communication link between the psychiatrist and patient.	Consists of pre-recorded videos of psychiatrist that are then sent to the patient to be viewed on their own time.
Allows for real-time live interaction and assessment.	Involves acquiring medical data and transmitting it to the psychiatrist at a convenient time for assessment offline.
Can gain additional details essential for care during the session, which is more efficient in making a clinical decision and providing advice during the interaction.	Patients can get timely care without needing to travel beyond the location of their primary care providers.
Real-time telepsychiatry provides timely care, especially in emergencies or urgent situations, and maintains the concept of the doctor-patient relationship by enabling a face-to-face analysis and treatment.	Wait times for specialty care are lessened, especially in areas with shortages of psychiatrists.
The doctor can see the patient and engage in conversation, much as they would in an office visit.	The store-and-forward process can overcome language and cultural barriers.
	Patients who are traveling or live in a different timezone can receive care without inconvenience.
	The turnaround time to receive answers to patient concerns/questions may be longer than synchronous telepsychiatry.

Although telepsychiatry is relatively new, it has already exhibited considerable success in both its effectiveness at treating psychiatric conditions as well as widespread corollary benefits. Although there are a few cases in which no significant improvement was seen between in-person psychiatry and telepsychiatry (five out of the 16 studies listed in Table 4), peripheral consequences such as decreased cost, better patient-doctor interactions, and higher satisfaction make telepsychiatry a viable, if not preferable choice to traditional in-person medicine. This, of course, only holds true for visits and disorders that do not necessitate physical examinations, which would be much more difficult to conduct virtually. Table 4 contains a comprehensive list of studies examining the effectiveness of telepsychiatry across multiple conditions, from depression and anxiety to dementia and post-traumatic illness, among others. 

**Table 4:** Studies showing the effectiveness of telepsychiatry and telemedicine before the COVID-19 pandemic RCT - Randomized controlled trials; STP - Synchronous telepsychiatry (Synchronous refers to the delivery of health information in real-time); ATP - Asynchronous telepsychiatry (Asynchronous refers to the “store-and-forward” technique, whereas a patient or physician collects medical history, images, and pathology reports and then sends it to a specialist physician for diagnostic and treatment expertise); T-CSCT - telepsychiatry-based culturally sensitive collaborative treatment; TP - Telepsychiatry; TAU - Treatment as usual; VCF - Videoconference; FTF - Face to face; SR – same room; WEB - Web-based tools; VCTP - Videoconference Telepsychiatry; CGI - Clinical global impressions; PCP - Primary care providers; HDRS - Hamilton depression rating scale; SCL-90 - Symptom Checklist-90; ADHD - Attention deficit hyperactivity disorder; ODD - oppositional defiant disorder; VADRS - Vanderbilt ADHD Rating Scale

Author, Country, Year	Type of Study	Psychiatric condition studied	Sample Size	Outcomes Measured	Results	Interpretation of study
Yellowlees et al, USA 2018 [27]	RCT (Asynchronous or ATP vs Synchronous or STP Telepsychiatry)	Depression, anxiety and substance abuse	ATP=77, STP= 81	1.Clinical outcome using Clinical global impressions (CGI) 2. Satisfaction 3. economic data	1.Significant improvement in CGI from baseline 2.Less average wait times (all for ATP) 3.More efficient use of psychiatrist time 4.Good teamwork along with PCP	1.Improved patient satisfaction 2.Relieve Physician burnout and well-being 3.Decrease time to essential mental health care
Farabee et al, USA, 2016 [29]	RCT (TP vs TAU)		N= 104	1.Satisfaction with treatment 2. Therapeutic alliance 3. Medication adherences 4. Psychological functioning.	1.High satisfaction with telepsychiatry 2.No significant group differences in medication adherence or psychological functioning. 3.Lower levels of the therapeutic alliance at follow-up.	1.Improved satisfaction with telepsychiatry
Xiong et al, USA 2018 [30]	RCT (STP vs ATP)	Dementia, depression	Total = 43, ATP=22, STP=21	Clinical global impressions (CGI) severity at 6month and 12 month	Significant improvement in CGI from baseline to 6-month follow-up, regardless of group assignment, Demonstrating the acceptability, feasibility, and impact of both forms of telepsychiatry in the Small nursing facility (SNF) setting.	1.Telepsychiatry is effective 2.Improved satisfaction rates with telepsychiatry
Shulman et al, USA, 2017 [31]	RCT (TP vs TAU)		N=22	Visit adherence	Not much difference in visit adherence between the telepsychiatry and treatment-as-usual groups (14%, compared with 15%). A greater number of participants in the telepsychiatry group reported less subjective difficulty in keeping appointments.	1.Increased compliance with telepsychiatry
O’reilly et al, Canada, 2007 [32]	RCT (TP vs face to face)		254 (face to face) 241(TP)	Distress from psychiatric symptoms using the Brief Symptom Inventory.	Psychiatric consultation and follow-up delivered by telepsychiatry produced clinical outcomes that were equivalent to those achieved when the service was provided face to face	Not much difference observed between telepsychiatry and in-person visits.
Zou et al, USA, 2016 [33]	RCT (T-CSCT vs TAU)	MDD	(N = 190), T-CSCT (n = 97), TAU (n = 93)	17-item Hamilton Depression Rating Scale (HDRS₁₇); the positive response was defined as a ≥ 50% decrease in HDRS₁₇ score	1. Response and remission were significantly greater for the T-CSCT group compared to the control group (odds ratio [OR] = 3.9 [95% CI, 1.9 to 7.8] and 4.4 [95% CI, 1.9 to 9.9], respectively). 2.T-CSCT group had significantly greater improvement over time in HDRS₁₇ (F4,95 = 4.59, P = .002), CGI-S (F4,95 = 4.22, P = .003), and CGI-I (F4,95 = 2.95, P = .02) scores.	Improved response rates with telepsychiatry
Chong et al, USA, 2012 [34]	RCT (WEB vs TAU)	Depression	N=167, WEB=80, TAU= 87	1.Acceptability 2.visit satisfaction 3. Antidepressant use. 4.Feasibility	1.WEB patients did not differ in the proportion of completed primary care versus telepsychiatry appointments and rated their working alliance with the psychiatrist and their visit satisfaction significantly higher than the TAU patients with their provider. Significantly more WEB than TAU patients used antidepressants.	1. Improved acceptability, feasibility, and satisfaction rates with telepsychiatry
De Las Cuevas et al, Spain, 2006 [35]	RCT (VCTP vs FTF)		N= 140	Clinical Global Impressions-Severity of Illness (CGI-S) and -Improvement (CGI-I) scales	1.Significant improvements were found on the CGI and SCL-90- R Global Indexes scores of both treatment groups, showing clear clinical state improvement. 2. statistically significant differences were observed when the efficacy of VCTP treatment was compared to FTF psychiatric treatment efficacy	Higher response rates and satisfaction rates in the telepsychiatry group.
Frueh et al, USA, 2007 [36]	RCT (TP vs SR)	Post-Traumatic Stress disorder	N=38, 21-SR, 17-TP	12-item SER-TACP (Therapist adherence and competence of treatment) was used to assess adherence and competence.	Findings suggest that therapist competence and adherence to cognitive-behavioural therapy is similar whether the treatment is delivered via TP or by traditional means, and TP does not compromise therapists’ ability to effectively structure sessions or build rapport with patients	1.Treatment Adherence and competence is similar in telepsychiatry vs in-person visits. 2. Improved patient-doctor interactions.
Ruskin et al, USA, 2004 [37]	RCT (TP vs in person)	Depression	N=118	1.Treatment response. 2. Treatment adherence. 3. Patient satisfaction. 4. Psychiatrist satisfaction. 5. Resource consumption or cost effects.	1. Hamilton Depression Rating Scale and Beck Depression Inventory scores improved over the treatment period and did not differ between treatment groups. 2. The two groups were equally adherent to appointments and medication treatment. No between-group differences in dropout rates or patients’ ratings of satisfaction with treatment were found. 3. Telepsychiatry was more expensive per treatment session, but this difference disappeared if the costs of psychiatrists’ travel to remote clinics more than 22 miles away from the medical center were considered.	1. Not much significant between treatment and in person groups. 2. Cost effective for clinics far away from the medical centers.
Moreno et al, USA, 2012 [38]	RCT (WEB vs TAU)	Depression	N=167	1. Montgomery-Åsberg Depression Rating Scale by clinician’s blind to treatment group and 2. Self-ratings on the nine-item Patient Health Questionnaire. 3. Quality of Life Enjoyment and Satisfaction Questionnaire. 4. Sheehan Disability Scale.	1. On all four measures, a significant interaction of time by intervention favoring the Webcam group was noted. 2. Results suggest that telepsychiatry delivered through the internet utilizing commercially available domestic Webcams and standard internet and computer equipment is effective and acceptable	Telepsychiatry was effective and acceptable.
Bishop et al, Canada, 2002 [39]	RCT (VCTP vs FTF)		N=24	Patient satisfaction with the services was assessed using the Client Satisfaction Questionnaire (CSQ-8), completed four months after the initial consultation	The mean scores were 25.3 in the FTF group and 21.6 in the videoconferencing group. Although there was a trend in favor of the FTF service, the difference was not significant	Not much significance in the telepsychiatry vs face to face visits groups.
Hulsbosch et al, Netherlands, 2017 [40]	RCT (VCF vs care as usual)	Severe mental illness	N=93, VCF 47, care as usual (CAU)= 46	Patient satisfaction was the primary outcome of the study. The ‘GGZ Thermometer’, a validated Dutch instrument for assessing patient satisfaction within the mental health care setting,11 was used	A statistically significant time by treatment interaction effect was found, where higher degree of satisfaction was associated with the patients in the VCF-group.	Improves patient satisfaction in the telepsychiatry group.
Maieritsch et al, USA, 2016 [41]	RCT (VCTP vs in person)	Post-Traumatic stress disorder (PTSD)	N=90	Clinician-administered PTSD scale (CAPS) scores	A trend was observed which suggested that Cognitive processing therapy (CPT) over VTC may be equivalent to the treatment delivered in person	Not much significance in the telepsychiatry vs in person group.
Myers et al, USA, 2015 [42]	RCT (VCF vs in-person)	Attention deficit hyperactivity disorder	N=223	Outcomes were diagnostic criteria for ADHD and ODD and role performance on the VADRS completed by caregivers (VADRS-Caregivers) and teachers (VADRS-Teachers) and impairment on the Columbia Impairment Scale-Parent Version (CIS-P)	Children assigned to the telehealth service model improved significantly more than children in the augmented primary care arm for VADRS-Caregiver criteria for inattention (χ(2)[4] = 19.47, p < .001), hyperactivity (χ(2)[4] = 11.91, p = .02), combined ADHD (χ(2)[4] = 14.90, p = .005), ODD (χ(2)[4] = 10.05, p = .04), and VADRS-Caregiver role performance (χ(2) [4] = 12.40, p = .01) and CIS-P impairment (χ(2)[4] = 20.52, p < .001).	Improved response rates in telepsychiatry group.
Hur et al, Republic of Korea, 2017 [43]	RCT - scenario-based CBT mobile app group, the Todac Todac group (TT group) and an app-based mood charting group (control group)	Depressive disorder	N=34	Outcomes measured using Dysfunctional attitude score (DAS), Beck’s depression inventory Scores(BDI-II) and Situation dependent trait version of state trait Anxiety inventory (STAI- X2)	1.After 3 weeks TT group showed lower DAS scores and significantly lower STAI-X2 scores compared to the control group. 2.Both groups showed reduced BDI-II scores.	Mobile based apps are helpful in delivering effective and feasible cognitive therapy.

Up until this point, we have discussed telepsychiatry in the context of virtual appointments and interactions that presumably take over phone or video calls, but with the advent of smartphones, mobile applications (apps) have become an area of interest. In Table 5, though, we have explored different mobile application interventions that are available for the sole purpose of both monitoring/assessing patients and/or providing treatment. The scope of conditions that these apps address is broad, ranging from anxiety disorders to schizophrenia to depression, etc. All studies reported high user satisfaction and a few even saw significant improvement in symptom reduction [44-51]. These findings, although preliminary, suggest a promising future for mobile applications that patients can use on their own time either independently or in conjunction with psychiatrist appointments and monitoring. 

**Table 5:** Tools utilized for telepsychiatry PDA - Personal digital assistants; VTC - Video teleconferencing; MoCA - Montreal Cognitive Assessment Tool; CBT - Cognitive behavioural therapy; VR-CBT - Virtual reality equipment for CBT; dCBT - Digital cognitive behavioural therapy; DSM-4 - Diagnostic and Statistical Manual of Mental Disorders 4th Edition; MDD - Major depressive disorder

Author, Country, Year	Tools	Study Design	Psychiatric Condition Studied	Description	Usefulness of Tool
De Sa & Carrico, USA, 2012 [44]	Mobile App Device: PDA	Description of app design process and the results of a qualitative exploratory trial study, utilizing direct observation, interviews, and questionnaires. Participants underwent 1 session.	Children with anxiety disorders	The existence of therapeutic treatment tools for young patients is low. Paper presents mobile apps that aim to help children (5-14 years of age) better quantify their fears through the use of drawing and components that improve patient intractability and understanding. The app was also designed to better support therapy procedures.	Out of 8 children interviewed: 3 were pleased with the experience, 5 found it easy to use, 7 found it easier than the paper equivalent. Both patients and therapists were satisfied and excited about the app.
Iiboshi et al., Japan, 2020 [45]	VTC-administered Montreal Cognitive Assessment Tool (MoCA)	Participants older than 60 years of age with and without cognitive impairment (mild cognitive impairment [MCI], dementia, and healthy controls [HC]) assessed using VTC and face-to-face (FTF)-administered MoCA at an interval of >2 weeks and <3 months.	Mild cognitive impairment (MCI) and dementia	Despite the various potential benefits of utilizing VTC-administered MoCA to detect and diagnose cognitive decline, not many studies have examined the reliability of such a tool, which was the aim of this study.	Intraclass correlation coefficient (ICC) for the MoCA was high in the entire sample overall, and moderate to high for the subgroups, indicating good agreement between VTC- and FTF-administration. Participants also indicated overall satisfaction with VTC-administered MoCA.
Graham et al., USA, 2020 [46]	IntelliCare: a suite of mobile apps providing a library of psychoeducation, and administering weekly symptom assessment	A two-arm randomized clinical trial with 146 patients, evaluating the efficacy of a mobile platform in providing treatment for depression and anxiety	Depression (positive on Patient Health Questionnaire-8; score>=10) and anxiety (Generalized Anxiety Disorder-7; scores >= 8)	Participants in intervention conditions began IntelliCare through onboarding calls with their coach. Coaches managed participants using an online dashboard, containing info on patient app use and their symptom assessments. Primary outcomes were assessed with PHQ-9 and GAD-7 at baseline and the follow-up assessments.	Individuals in the intervention condition had a greater reduction in depression and anxiety symptoms compared with treatment-as-usual participants. These changes were also sustained over 2 months.
Donker et al., Netherlands, 2019 [47]	ZeroPhobia, a fully self-guided virtual reality equipment for CBT	Single-blind randomized clinical trial with 193 participants with acrophobia symptoms to examine the effectiveness of ZeroPhobia	Acrophobia (score of at least 45.45 on Acrophobia Questionnaire (AQ)-Anxiety)	Participants received 6 animated CBT-based modules that took between 5-40 minutes and were asked to complete it within 3 weeks. The primary outcome was AQ scores.	Intervention through ZeroPhobia showed a significant reduction in acrophobia symptoms compared with control on AQ scores. The VR-CBT app was also rated as user friendly, showing the ease of using technology-based interventions.
Espie et al., United Kingdom, 2019 [48]	dCBT for insomnia	An online, 2-arm, parallel-group randomized trial comparing dCBT for insomnia with sleep hygiene education (SHE) evaluated 1711 participants with self-reported insomnia symptoms	Insomnia	dCBT was delivered to participants using the web and/or mobile channels along with treatment as usual. SHE was a website and a downloadable booklet along with treatment as usual. Primary outcomes were scores on the Patient-Reported Outcomes Measurement Information System: Global Health Scale, Warwick-Edinburgh Mental Well-being Scale, and Glasgow Sleep Impact Index.	The use of dCBT was associated with a small improvement in functional health, and psychological well-being, and a large improvement in sleep-related quality of life. A large improvement in insomnia was the mediator of these outcomes. Thus, we see that dCBT is effective in reducing insomnia symptoms, which mediates the aforementioned effects.
Ainsworth et al., United Kingdom, 2013 [49]	Android app “ClinTouch” for monitoring schizophrenic patients	Randomized control trial with 24 participants comparing two monitoring systems for schizophrenic patients — one that is app-based and one that utilizes is short messaging service-based.	Schizophrenia (DSM-4)	Schizophrenic participants were randomly allocated to completing 6 days of assessment, involving four sets of questions a day, using the smartphone app or SMS text-only implementation. A 1-week break was given before the alternative method was used for 6 more days. Feedback was obtained at the end of each period of sampling.	A significantly larger proportion of data was completed through the smartphone application compared to the short-messaging implementation. Most participants also reported a preference for the app, citing its ease of use.
Macias et al., USA, 2015 [50]	iPhone and Android app “WellWave” designed to facilitate self-directed healthy living and psychiatric recovery.	Pilot study that tested the usability and acceptability of WellWave for adults diagnosed with psychiatric conditions, with the focus on walking as a physical exercise.	A wide variety of psychiatric disorders (schizophrenia, bipolar disorder, or MDD)	Eleven volunteers, each with at least one of the mentioned psychiatric (4 schizophrenics) completed an individual WellWave training session before completing a 4-week pilot. 9 participants provided pretest and posttest questionnaire data.	Participants used the app an average of 94% of the days and 70% of participants went on a walk at least 2x a week. All participants reported being satisfied with the app, reporting feelings of well-being as well as practice benefits.
Ben-Zeev et al., USA, 2014 [51]	Android app “FOCUS” designed for real-time illness management of schizophrenic patients	Single-arm feasibility trial examining the efficacy and acceptability of real-time/real-place illness management to support schizophrenic patients	Schizophrenia or schizoaffective disorder	33 individuals w/schizophrenia or schizoaffective disorders used the FOCUS app for 1 month, with 32 users completing the trial fully.	Completers used FOCUS on 86.5 % of days for an average of 5.2x a day. 90% of participants rated the app as highly acceptable and usable. Study demonstrated the feasibility, acceptability, and preliminary efficacy of the FOCUS intervention.

Unfortunately, there is very limited literature on the current implementation of telepsychiatry during the coronavirus pandemic (Table 6). This is not a reflection of the inefficacy of telepsychiatry, though, but rather a reflection of its underutilization. This is a field of untapped potential that needs to be further explored and expanded upon, especially with the current climate of the pandemic. As promising and beneficial telepsychiatry may seem, though, it is necessary to recognize that no telemedicine program can be created overnight. There are challenges involved in adapting to this new method of medicine. Table 2 above highlights barriers to general telepsychiatry-the most prominent ones being the need for technological literacy on both the physician and patient end, as well the possible negative effects of eliminating the in-person patient-doctor interaction that is often used to build rapport. 

**Table 6:** Utilization of telepsychiatry during COVID-19 pandemic: utilization, advantages, and challenges

Higher utilization rate of Telepsychiatry						
Author, Country, Year	Type of Study	Psychiatric condition studied	Sample Size	Outcomes Measured	Results	Interpretation of study
Zarghami et al., Iran, 2020 [52]	Report in which video chat was used	Insomnia seen in 24 (29.3%) patients and adjustment disorder in 13 (15.9%) patients	Total(82) 32 inpatient, 50 outpatient, 32 male and 50 female	Patient Health Questionnaire-9 (PHQ-9), Generalized Anxiety Disorder Assessment (GAD-7), and Perceived Stress Scale-14 (PSS 14) questionnaires	Female and hospitalized patients presented significantly more frequent comorbidities than males and outpatients	Telepsychiatry in the early stages of mental problems during a catastrophic event like the coronavirus pandemic, can be an efficient instrument for the screening of psychosomatic comorbidities, so that pharmacological treatment (considering possible drug interactions with COVID-19 medications) and psychotherapeutic intervention can be optimized by psychiatrists.
Chen et al, USA, 2020 [53]	A review of key changes that were implemented during COVID-19 with regards to psychiatric care, as well as observations of advantages and limitations to telepsychiatry	N/A	N/A	N/A	Biggest advantage of telepsychiatry during COVID-19 pandemic is limiting viral transmission and reducing risk of exposure. Clinical benefits include decreased no-show rates, increased ease of scheduling and access to care, among other advantages. The largest limitations included the lack of a physical examination and mental status markers, loss of intimacy, and exacerbated disparities for those who lack access to technology.	
Johns Hopkins Medicine, 2020 [54]	Description of transitions to telepsychiatry at Johns Hopkins Hospital during the COVID-19 pandemic	N/A	N/A	N/A	Patients with opioid use disorder have been able to benefit from at-home video consultation and management through telepsychiatry.	The efficacy and benefits of telepsychiatry during the coronavirus pandemic have been preliminarily demonstrated at a large scale hospital (i.e., Johns Hopkins), implicating the clinical benefits of transitioning to telepsychiatry.

Specific to COVID-19, structured interviews with clinicians revealed problems that they ran into when transitioning to telepsychiatry (Table 7). These included decreased clinical data available to make an assessment or diagnosis and technological challenges (lack of reliable access to a computer, smartphone, or the internet) among other issues.

**Table 7:** Challenges in maintaining telepsychiatry after COVID-19 pandemic OUD - Opioid use disorder

Author/Year	Type of Study	Psychiatric condition studied	Sample Sizes	Outcomes Measured	Results	Interpretation of study
Uscher-Pines et al., USA, 2020 [55]	Qualitative Semi structured interview	N/A	N/A 20 outpatient psychiatrists from 5 US states semi structured interviews	N/A	Interview summary Matrix analysis	1. Challenges affecting the quality of provider-patient interactions 2. Decreased clinical data for assessment, 3. Diminished patient privacy 4. Increased distractions in the patient's home setting. 5.Disadvantaged patients lacked reliable access to a computer, smartphone, and the internet.
Uscher-Pines et al., USA, 2020 [56]	Semi structured interview	OUD	18 clinicians from 10 states	Changes included waiving urine toxicology screening, sending patients home with a larger supply of OUD medications, and requiring fewer visits	Interview summary	1. Less structure and accountability, 2. Less information to inform clinical decision-making, 3. Challenges in establishing a connection, 4. Technological challenges, and shorter visits.

Given this, it is necessary to implement some advanced directives that will smoothly aid the shift toward telepsychiatry. We provide a few recommendations below. First and foremost, it will be crucial to evaluate telehealth competency among psychiatrists before establishing a standard for telepsychiatry training, in addition to creating frameworks for virtual psychiatric practice. This first step will provide a stable foundation for the entire psychiatric field to promote quality and regulated care.

Advance Directives

The following recommendations may be beneficial in improving telepsychiatry and telehealth services. Conducting and evaluating current telehealth competency among psychiatrists and physicians to promote quality care across practices [57]. Evaluate and improve current frameworks for technological competency and use in psychiatry [58]. Establish standardized training for psychiatrists who are currently practicing in the field extend telepsychiatry training to include undergraduate medical education for early training and introduction to telepsychiatry [59]. Provide patients with technological training to ensure that they have the digital literacy to use these virtual services and apps [60, 61]. Work with social workers to ensure that patients in remote areas have consistent access to technological resources.

## Conclusions

Overall, the use of telemedicine to provide medical care is not novel, but the COVID-19 pandemic has forced the US healthcare system to promptly shift to telehealth, whether or not it was ready to do so. When looking at this shift to telemedicine, some specialities have been focused on greater than others, and psychiatry is one field that has been overlooked. This current review examined the feasibility, efficacy, and likelihood of successful implementation of telepsychiatry both during the pandemic and beyond. The advantageous benefits of telepsychiatry, whether it be used in conjunction with mobile smartphone applications or synchronous/asynchronous, are widespread and many. The potential of using virtual medicine to change the way the psychiatric field is structured is unbounded, although this transition is not without barriers or difficulties. To address these issues, preemptive steps must be taken to standardize practice and thoroughly prepare healthcare providers. With careful planning and collaborative teamwork, telepsychiatry implementation can provide practical and satisfactory delivery of mental healthcare, benefitting both patients and clinicians.

## Appendices

Jayasudha Gude and Rashmi V. Subhedar work as equal authors.

Authors' contributions: Conceptualization: Jayasudha Gude and Rashmi V. Subhedar; Acquisition of literature: Michelle H. Zhang, Pratik Jain, Jatminderpal Bhela; Formal investigation: Jayasudha Gude, Rashmi V. Subhedar; Writing - original draft preparation: Jayasudha Gude, Rashmi V. Subhedar, Fariha Bangash, Nikhila Veluri, Ya-Ching Hsieh​​​; Writing - review, critical feedback, and editing: Batool Z. Sheikh, Mansi R. Shah, Kapil Aedma, Urvish K. Patel, Tapan Parikh; Supervision: Kapil Aedma,Tapan Parikh​​​​​​.
